# Sharp upper bound for anisotropic Rényi entropy and Heisenberg uncertainty principle

**DOI:** 10.1007/s00229-026-01694-7

**Published:** 2026-03-14

**Authors:** Marianna Chatzakou, Michael Ruzhansky, Anjali Shriwastawa

**Affiliations:** 1 Department of Mathematics: Analysis, Logic and Discrete Mathematics, Ghent University, Krijgslaan 281, B 9000 Ghent, Belgium; 2 School of Mathematical Sciences, Queen Mary University of London, Mile End Road, E1 4NS London, United Kingdom; 3 DST-Centre for Interdisciplinary Mathematical Sciences, Organization, Banaras Hindu University, 221005 Varanasi, India

**Keywords:** 94A17, 22E30, 35H10

## Abstract

In this paper, we prove the anisotropic Shannon inequality for the Rényi entropy with the best constant on Folland-Stein homogeneous Lie groups. As a consequence, we also prove the optimal Shannon inequality in the same setting. Using a logarithmic Sobolev inequality in the setting of stratified groups, we prove a Heisenberg-type uncertainty principle in the latter setting.

## Introduction

Shannon and Rényi entropies were initially served as characteristics of probability distributions. To begin with, the Shannon entropy was introduced by Shannon in [20], and for a discrete random variable *X* with distribution *p*(*x*) it is given via

$$ h[X]=-\sum _i p(x_i)\log p(x_i)\,. $$

In the continuous setting for a continuous random variable *X* with (probability) density *u*(*x*), the natural analog of the previous definition is as follows:

1 $$\begin{aligned} h[X]:=h[u]=-\int _{\mathbb {R}^n}u(x)\log u(x)\,dx\,. \end{aligned}$$

Clearly the Shannon entropy (1) makes sense for a nonnegative $u \in L^1(\mathbb {R}^n)$ such that $\Vert u\Vert _{L^1(\mathbb {R}^n)}=1$. In the sequel we will write *h*[*u*] instead of *h*(*X*), to denote the Shannon entropy of the continuous random variable *X* with density *u*. Let us point out that in the continuous case that we study here, *p*(*x*) is not a probability, but a probability density, and the latter are not the same. Indeed, the quantity *h*[*X*], for *X* being a discrete random variable, is always non-positive, while if *X* is the continuous random variable which is uniformly distributed over the interval (*a*, *b*); that is

$$\begin{aligned} u(x)=\left\{ \begin{array}{ll} \frac{1}{b-a}\,,\quad \text {if}\quad x \in (a,b)\\ 0\,,\quad \quad \text {otherwise}\,, \end{array}\right. \end{aligned}$$

then $h[X]=h[u]=-\log (b-a)$, and clearly $h[u]>0$ if $b-a<1$, see e.g. [17]. Therefore, in the continuous case, one cannot, strictly speaking, talk about the “amount of information” represented by the entropy.

There are other features of the Shannon continuous entropy that make its study a quite involved subject; see for example [13]. Despite these difficulties, continuous informatics is a field of wide interest, c.f. the monograph of Ihara [10], or the work of Conrad [6]. Particularly, in relation to partial differential equations, the conclusion of the Boltzman H-Theorem, see e.g. [25], holds true for the solution to the heat equation on $\mathbb {R}^n$

2 $$\begin{aligned} \partial _t u=\Delta u\,, \quad t>0\,; \end{aligned}$$

that is the Shannon entropy (1) is not decreasing in time, since

$$ \frac{d}{dt}h[u(x,t)]=\int _{\mathbb {R}^n}\frac{1}{u(x,t)}|\nabla u(x,t)|^2\, dx \ge 0\,. $$

Analogously, the Rényi entropy, introduced by Rényi in [16], is for nonnegative function $u \ge 0$ with $\Vert u\Vert _{L^1(\mathbb {R}^n)}=1$, given by

3 $$\begin{aligned} h_\alpha [u]=\frac{1}{1-\alpha } \log \int _{\mathbb {R}^n}u(x)^\alpha \,dx\,, \end{aligned}$$

“corresponds” to the nonlinear diffusion equations

$$ \partial _t u= \Delta u^\alpha \,,\quad t>0, \alpha >0, \alpha \ne 1\,. $$

It is easy to check that the Rényi entropy (3) is an extension of the Shannon entropy (1) for different values of $\alpha$ in the sense that

$$ \lim _{\alpha \rightarrow 1}h_{\alpha }[u]=h[u]\,, $$

for any function *u* as above. Both Shannon and Rényi entropies, in both discrete and continuous settings, are monotone in the sense that they increase whenever an independent random variable is added, and hence a natural question is whether one can find an upper bound for them; in the Euclidean setting $\mathbb {R}^n$, this is given by Shannon’s inequality [20, 22] which is an upper bound of the form:

4 $$\begin{aligned} h[u] \le \frac{n}{2} \log \left( \frac{2 \pi e}{n} \int _{\mathbb {R}^n}|x|^2 u(x)\,dx \right) \,. \end{aligned}$$

Hence inequality (4) shows that the Shannon entropy of a function *u* is bounded by the second moment of *u*, and the constant $\frac{2\pi e}{n}$ is the best possible. In the case of the Rényi entropy, this upper bound reads as follows:

5 $$\begin{aligned} h_{\alpha }[u]\le \frac{n}{b} \log \left( C_b \int _{\mathbb {R}^n}|x|^b u(x)\,dx \right) \,,\quad \alpha \in \mathbb {R}^{+}\setminus \{1\}\,; \end{aligned}$$

that is the Rényi entropy of the function *u* is bounded by the $b^{\text {th}}$-moment of *u*, where $b>0$ depends on the range of $\alpha$. Both inequalities (4) and (5) are valid for functions *u* in some suitable weighted Lebesgue spaces. Inequality (5) is sharp, and the constant $C_b$ in (5) depends on the parameter *b* on the right-hand side of (5), which in turn depends on the parameter $\alpha$ and is given explicitly in [22] where the aforementioned inequality is proved. For the so-called Shannon inequality in the Euclidean setting (4) we refer to the paper [15], while Shannon’s inequality has also been proved in the general setting of homogeneous Lie groups in [3]. Properties of both entropies have been widely studied; see e.g. [1] and references therein.

Applications of Shannon’s inequality (4) and Shannon’s inequality for the Rényi entropy also include the positivity of the relative Lyapunov functional

$$ H_\alpha [u|v]=H_\alpha [u]-H_\alpha [v]\,, $$

where $H_\alpha [\cdot ]$ is the Lyapunov functional, see e.g. [14], given by

$$ H_\alpha [u]=\frac{1}{\alpha -1}\int _{\mathbb {R}^{n}}u(x)^\alpha \,dx +\frac{1}{2}\int _{\mathbb {R}^{n}}|x|^2 u(x)\,dx\,, $$

see [22] and [24]. Importantly, the combination of Shannon inequality (4) together with the version of logarithmic Sobolev inequality thanks to Stam [21]

6 $$\begin{aligned} h[u] \ge -\frac{n}{2} \log \left( \frac{1}{2 n \pi e}\int _{\mathbb {R}^n} \frac{1}{u(x)}|\nabla u(x)|^2\,dx \right) \,, \end{aligned}$$

which holds true for non-negative $u \in L^1(\mathbb {R}^n)$ with $u^{1/2} \in H^1(\mathbb {R}^n)$, gives rise to the Heisenberg uncertainty principle; that is for such *u* we have

7 $$\begin{aligned} n\le \left( \int _{\mathbb {R}^n}|x|^2 u(x)\,dx \right) ^{\frac{1}{2}}\left( \int _{\mathbb {R}^n}\frac{1}{u(x)}|\nabla u(x)|^2\,dx \right) ^{\frac{1}{2}}\,, \end{aligned}$$

where *n* is the best possible. Still in the Euclidean setting, Carillo and Toscani [2] proved the logarithmic Sobolev-type inequalities for the Rényi entropy; see also [19].

The setting of the current work is that of homogeneous Lie groups introduced in Section 2. In this setting, in [3] the authors proved the (anisotropic) Shannon inequality, with an explicit constant that is sharp, see the discussion that follows after Remark 3.3. Here we prove the analogue of Shannon’s inequality for the Rényi entropy as in (5) for the setting of homogeneous Lie groups, see Theorem 3. For the proof of the latter, we follow the lines in [22] and we prove the aforementioned inequality with the best constant as it happens in [22], see Corollary 3.2. Later on, in Theorem 3.4, we prove the Shannon inequality as in (5) in the setting of homogeneous Lie groups, using the fact that the Shannon entropy is the limiting case when $\alpha \rightarrow 1$ of the Rényi entropy. The Shannon inequality in the aforesaid setting was also proved in [3] using different methods and the constant there coincides with ours since both are optimal. Our final result is the Heisenberg uncertainty principle in the setting of stratified Lie groups, see Corollary 3.7. The latter is proved by combining Shannon’s inequality with a version of logarithmic Sobolev inequality in the aforesaid setting that is derived by the logarithmic Sobolev inequality as in [4]; see Theorem 3.6.

## Preliminaries

In this section, we give a brief description of our setting of homogeneous Lie groups. Such groups were initiated by Folland and Stein, see [8], and later on, they became a subject of study by many authors; see [7] and references therein. The notation that we adopt in the sequel follows the more recent open access monograph on homogeneous Lie groups [7].

A connected, simply connected Lie group $\mathbb {G}\cong \mathbb {R}^N$ whose Lie algebra $\mathfrak {g}$ admits a gradation of the form

8 $$\begin{aligned} \mathfrak {g}=\oplus _{j=1}^{\infty }V_{j}\,,\end{aligned}$$

where finitely many $V_j$’s are nonzero and satisfy relations of the form $[V_{i},V_{j}]\subset V_{i+j}$ is called a graded Lie group. Graded Lie groups are naturally homogeneous Lie groups meaning that there exists a dilation mapping denoted as $D_\lambda : \mathbb {R}^N \rightarrow \mathbb {R}^N$, where $\lambda > 0$, that is an automorphism of $\mathbb {G}$, and so also of $\mathfrak {g}$. Particularly, for $\lambda >0$, the mapping $D_\lambda$ acts on $x \in \mathbb {G}$ via

9 $$\begin{aligned} D_\lambda (x)= (\lambda ^{v_1}x_1,\lambda ^{v_2}x_2,\ldots ,\lambda ^{v_N}x_N) ,\quad v_1,v_2,\dots ,v_N > 0\,. \end{aligned}$$

The so-called weights $v_1,\cdots , v_n$ determine the homogeneous dimension, usually denoted by *Q*, of the homogeneous group $\mathbb {G}$ in the following way:

$$\begin{aligned} Q=v_1+v_2+...+v_N. \end{aligned}$$

A homogeneous group is unimodular and the unique Haar measure denoted by *dx* on $\mathbb {G}$ is the Lebesgue measure on the underlying manifold $\mathbb {R}^N$. Hence for $\omega \subset \mathbb {G}$ being a measurable set in $\mathbb {G}$, if $|\omega |$ stands for the volume of $\omega$, then for $\lambda > 0$ we have

10 $$\begin{aligned} |D_\lambda (\omega )|=\lambda ^Q |\omega | \quad \text {and} \quad \int _{\mathbb {G}} f(D_\lambda (x)) dx = \lambda ^{-Q}\int _{\mathbb {G}} f(x) dx. \end{aligned}$$

For any homogeneous Lie group $\mathbb {G}$, there exists a homogeneous (with respect to the dilations determined by $D_\lambda$ on $\mathbb {G}$) quasi-norm $|\cdot |$ defined on $\mathbb {G}$; that is $|\cdot |:\mathbb {G} \rightarrow [0,\infty )$ is a continuous, non-negative function that satisfies the following conditions:

(i) For all $$x \in \mathbb {G}$$, $$|x|=|x^{-1}|$$.
(ii) For all $x \in \mathbb {G}$ and $\lambda > 0$, $|\lambda x|=\lambda |x|$.
(iii) $|x|=0$ if and only if $x=0$.

We note that in the sequel we use the notation $|\cdot |$ to denote both the volume of a measurable set in $\mathbb {G}$ and the homogeneous quasi-norm of an element $x \in \mathbb {G}$, and the meaning of $|\cdot |$ is each appearance will be clear from the context.

Finally, let us introduce the following Lebesgue spaces that are useful for our purposes: For $a>0$, the weighted Lebesgue space denoted by $L^{1}_{a}(\mathbb {G})$ is defined as follows:

$$ L_{a}^{1}(\mathbb {G})=\{u \in L^{1}(\mathbb {G}) \quad \text {such that}\quad |x|^{a}u\in L^1(\mathbb {G}) \}\,, $$

where |*x*| stands for the quasi-norm of $x \in \mathbb {G}$.

## Main Results

In this section, we establish the main results of the paper starting with proving the Shannon inequality for the Rényi entropy in the setting of homogeneous Lie groups.

### Theorem 3.1

Let $\mathbb {G}$ be a homogeneous Lie group of homogeneous dimension *Q*, and let $|\cdot |$ be a homogeneous quasi-norm on $\mathbb {G}$. Suppose that $\alpha >0$, $\alpha \ne 1$, and

$$\begin{aligned} b> \left\{ \begin{array}{ll} Q\left( \frac{1}{\alpha }-1 \right) \,,\quad & \text {if}\quad 0<\alpha <1,\\ 0\,, \quad & \text {if}\quad \alpha >1. \end{array}\right. \end{aligned}$$

Then, for any nonnegative function $u \in L^1_b(\mathbb {G})$ with $\Vert u\Vert _{L^1(\mathbb {G})}=1$, the inequality

11 $$\begin{aligned} \frac{1}{1-\alpha } \log \int _\mathbb {G}u(x)^\alpha \, dx \le \frac{Q}{b} \log \left( A_{\alpha , Q,b} \int _\mathbb {G}|x|^b u(x) dx \right) , \end{aligned}$$

holds, where

$$\begin{aligned} A_{\alpha , Q,b}:=\left\{ \begin{array}{ll} \left( \frac{b}{|\mathfrak {S}|}\frac{\Gamma \left( \frac{1}{1-\alpha }\right) }{\Gamma \left( \frac{1}{1-\alpha }-\frac{Q}{b}\right) \Gamma (\frac{Q}{b})}\right) ^{-\frac{b}{Q}}\, \left( \frac{\alpha b}{\alpha b-Q(1-\alpha )}\right) ^{\frac{b}{Q(1-\alpha )}} \left( \frac{\alpha b-Q(1-\alpha )}{Q(1-\alpha )} \right) \,, & \text {if}\quad 0<\alpha <1,\\ \frac{\alpha b}{Q(\alpha -1)}\left( \frac{\alpha b +Q(\alpha -1)}{\alpha b} \right) ^{\frac{Q(\alpha -1)+b}{Q(\alpha -1)}} \left( \frac{b}{|\mathfrak {S}|}\frac{\Gamma \left( \frac{\alpha }{\alpha -1}+\frac{Q}{b}\right) }{\Gamma \left( \frac{Q}{b} \right) \Gamma \left( \frac{\alpha }{\alpha -1} \right) }\right) ^{-\frac{b}{Q}}\,,\quad & \text {if}\quad \alpha >1\,, \end{array}\right. \end{aligned}$$

and $|\mathfrak {S}|$ stands for the $Q-1$ dimensional surface measure of the unit (quasi-)sphere with respect to $|\cdot |.$

### Proof of Theorem 3.1

Note that it is enough to prove (11) for a nonnegative function $u \ge 0$ that is smooth on $\mathbb {G}$. The result will then follow by a density argument. We will treat the cases where $\alpha \in (0,1)$ and $\alpha >1$ separately. Let us first consider the case $\alpha \in (0,1)$. For such values of $\alpha$ and for $b>Q\left( \frac{1}{\alpha }-1\right)$ we consider the auxiliary function $\phi _1$ given by

$$\begin{aligned} \phi _1(x)=C_{1}\left( 1+|x|^b\right) ^{\frac{1}{\alpha -1}}\,, \end{aligned}$$

with $C_1$ computed in the Appendix, see (A6). Using Jensen’s inequality for the convex function $\log 1/t$ and the the probability measure $\frac{u(x)^{\alpha }}{\Vert u\Vert ^{\alpha }_{L^{\alpha }(\mathbb {G})}}dx$ we estimate from above the relative entropy of *u* and $\phi _1$ as follows

12 $$\begin{aligned} \begin{aligned} \int _{\mathbb {G}} u(x)^\alpha \log \frac{\phi _1(x)}{u(x)}dx&\ = \frac{\Vert u\Vert ^{\alpha }_{L^{\alpha }(\mathbb {G})}}{\alpha } \int _{\mathbb {G}} \frac{u(x)^{\alpha }}{\Vert u\Vert ^{\alpha }_{L^{\alpha }(\mathbb {G})}}\log \frac{\phi _1(x)^{\alpha }}{u(x)^{\alpha }}dx \\  &\le \frac{\Vert u\Vert ^{\alpha }_{L^{\alpha }(\mathbb {G})}}{\alpha } \log \left( \frac{1}{\Vert u\Vert ^{\alpha }_{L^{\alpha }(\mathbb {G})}} \int _{\mathbb {G}} \phi _1(x)^{\alpha }\,dx\right) \\  &= \frac{\Vert u\Vert ^{\alpha }_{L^{\alpha }(\mathbb {G})}}{\alpha } \log \frac{\Vert \phi _1\Vert ^{\alpha }_{L^{\alpha }(\mathbb {G})}}{\Vert u\Vert ^{\alpha }_{L^{\alpha }(\mathbb {G})}}\,, \end{aligned} \end{aligned}$$

where $\Vert \phi _1\Vert ^{\alpha }_{L^{\alpha }(\mathbb {G})}$ is computed in Appendix, see (A11), as

$$ \Vert \phi _1\Vert ^{\alpha }_{L^{\alpha }(\mathbb {G})}=C_{1}^{\alpha -1} \frac{\alpha b }{\alpha b- Q(1-\alpha )}\,, $$

where $C_1$ is given in (A6).

Let us now give a lower bound for the relative entropy of *u* and $\phi _1$. By Jensen’s inequality for the same probability measure, and since $\alpha <1$, we have

13 $$\begin{aligned} \begin{aligned} \int _{\mathbb {G}}u(x)^{\alpha } \log \frac{\phi _1(x)}{u(x)}\,dx&\ = \frac{1}{\alpha -1} \int _{\mathbb {G}} \frac{\Vert u\Vert _{L^{\alpha }(\mathbb {G})}^{\alpha }}{\Vert u\Vert _{L^{\alpha }(\mathbb {G})}^{\alpha }} u(x)^{\alpha } \log \frac{1+|x|^b}{u(x)^{\alpha -1}}\,dx+\Vert u\Vert _{L^{\alpha }(\mathbb {G})}^{\alpha } \log C_1 \\  &\ge \frac{\Vert u\Vert _{L^{\alpha }(\mathbb {G})}^{\alpha }}{\alpha -1} \log \left( \frac{1}{\Vert u\Vert _{L^{\alpha }(\mathbb {G})}^{\alpha }}+\frac{1}{\Vert u\Vert _{L^{\alpha }(\mathbb {G})}^{\alpha }}\int _{\mathbb {G}}|x|^b u(x)\,dx\right) \\  &+\Vert u\Vert _{L^{\alpha }(\mathbb {G})}^{\alpha } \log C_1\,, \end{aligned} \end{aligned}$$

where we have used the fact that $\Vert u\Vert _{L^1(\mathbb {G})}=1$. A combination of (12) and (13) gives

14 $$\begin{aligned} \frac{1}{\alpha -1} \log \left( \frac{1}{\Vert u\Vert _{L^{\alpha }(\mathbb {G})}^{\alpha }}+\frac{1}{\Vert u\Vert _{L^{\alpha }(\mathbb {G})}^{\alpha }}\int _{\mathbb {G}}|x|^b u(x)\,dx \right) +\log C_1 \le \frac{1}{\alpha } \log \frac{\Vert \phi _1\Vert _{L^{\alpha }(\mathbb {G})}^{\alpha }}{\Vert u\Vert _{L^{\alpha }(\mathbb {G})}^{\alpha }}\,. \end{aligned}$$

Now, let us apply inequality (14) to the function $\tilde{u}(x):=\lambda ^{Q}u(D_\lambda (x))$. To this end, first we note that

15 $$\begin{aligned} \frac{1}{\Vert \tilde{u}\Vert _{L^{\alpha }(\mathbb {G})}^{\alpha }}+\frac{1}{\Vert \tilde{u}\Vert _{L^{\alpha }(\mathbb {G})}^{\alpha }}\int _{\mathbb {G}}|x|^b \tilde{u}(x)\,dx=\frac{\lambda ^{-Q(\alpha -1)}}{\Vert u\Vert _{L^{\alpha }(\mathbb {G})}^{\alpha }}+\frac{\lambda ^{-Q(\alpha -1)-b}}{\Vert u\Vert _{L^{\alpha }(\mathbb {G})}^{\alpha }}\int _{\mathbb {G}}|x|^b u(x)\,dx\,, \end{aligned}$$

since

$$\begin{aligned}  &   \Vert \tilde{u}\Vert _{L^{\alpha }(\mathbb {G})}^{\alpha } = \int _{\mathbb {G}}|\lambda ^{Q}u(D_\lambda (x))|^\alpha \,dx \\  &   {\mathop {=}\limits ^{y=D_\lambda (x)}} \int _{\mathbb {G}}\lambda ^{Q\alpha }|u(y)|^\alpha (\lambda ^{-Q}dy) \\= &   \lambda ^{Q(\alpha -1)}\Vert u\Vert ^{\alpha }_{L^{\alpha }(\mathbb {G})}. \end{aligned}$$

Similarly we have

16 $$\begin{aligned} \frac{1}{\alpha } \log \frac{\Vert \phi _1\Vert ^{\alpha }_{L^{\alpha }(\mathbb {G})}}{\Vert \tilde{u}\Vert ^{\alpha }_{L^{\alpha }(\mathbb {G})}}=\frac{1}{\alpha } \log \frac{\lambda ^{-Q(\alpha -1)}\Vert \phi _1\Vert ^{\alpha }_{L^{\alpha }(\mathbb {G})}}{\Vert u\Vert ^{\alpha }_{L^{\alpha }(\mathbb {G})}}=\frac{1}{\alpha -1} \log \frac{\lambda ^{-Q(\alpha -2+\frac{1}{\alpha })}\Vert \phi _1\Vert ^{\alpha -1}_{L^{\alpha }(\mathbb {G})}}{\Vert u\Vert ^{\alpha -1}_{L^{\alpha }(\mathbb {G})}}\,. \end{aligned}$$

Hence, with the use of (15) and (16), inequality (14) for the function $\tilde{u}$ reads as follows

$$\begin{aligned} \begin{aligned}&\ \frac{1}{\alpha -1} \log \left( \frac{\lambda ^{-Q(\alpha -1)}}{\Vert u\Vert _{L^{\alpha }(\mathbb {G})}^{\alpha }}+\frac{\lambda ^{-Q(\alpha -1)-b}}{\Vert u\Vert _{L^{\alpha }(\mathbb {G})}^{\alpha }}\int _{\mathbb {G}}|x|^b u(x)\,dx \right) +\log C_1 \\  &\le \frac{1}{\alpha -1} \log \frac{\lambda ^{-Q(\alpha -2+\frac{1}{\alpha })}\Vert \phi _1\Vert ^{\alpha -1}_{L^{\alpha }(\mathbb {G})}}{\Vert u\Vert ^{\alpha -1}_{L^{\alpha }(\mathbb {G})}} \,. \end{aligned} \end{aligned}$$

Using the properties of the logarithm, the latter inequality can be rearranged as follows

17 $$\begin{aligned} \begin{aligned}&\ \frac{1}{\alpha -1} \log \left( \lambda ^{Q\left( \frac{1}{\alpha }-1\right) }+\lambda ^{Q\left( \frac{1}{\alpha }-1 \right) -b}\int _{\mathbb {G}}|x|^b u(x)\,dx \right) \\  &\le \frac{1}{\alpha -1} \log \left( \frac{C_{1}^{1-\alpha } \Vert \phi _1\Vert ^{\alpha -1}_{L^{\alpha }(\mathbb {G})}}{\Vert u\Vert _{L^{\alpha }(\mathbb {G})}^{-1}} \right) \,, \end{aligned} \end{aligned}$$

and since

$$ \frac{1}{\alpha -1} \log \left( \frac{C_{1}^{1-\alpha } \Vert \phi _1\Vert ^{\alpha -1}_{L^{\alpha }(\mathbb {G})}}{\Vert u\Vert _{L^{\alpha }(\mathbb {G})}^{-1}} \right) =- \log \left( \frac{C_1}{\Vert \phi _1\Vert _{L^{\alpha }(\mathbb {G})}}\Vert u\Vert _{L^{\alpha }(\mathbb {G})}^{\frac{1}{1-\alpha }} \right) $$

multiplying (17) by $-1$ we get

18 $$\begin{aligned} \log \left( \frac{C_1}{\Vert \phi _1\Vert _{L^{\alpha }(\mathbb {G})}}\Vert u\Vert _{L^{\alpha }(\mathbb {G})}^{\frac{1}{1-\alpha }} \right) \le \frac{1}{1-\alpha } \log \left( \lambda ^{Q\left( \frac{1}{\alpha }-1\right) }+\lambda ^{Q\left( \frac{1}{\alpha }-1 \right) -b}\int _{\mathbb {G}}|x|^b u(x)\,dx \right) \,, \end{aligned}$$

where the last inequality holds true for any $\lambda >0$. In view of minimising the right-hand side of (18) over $\lambda >0$, we set

$$ M(\lambda ):= \lambda ^{Q\left( \frac{1}{\alpha }-1\right) }+\lambda ^{Q\left( \frac{1}{\alpha }-1 \right) -b}\int _{\mathbb {G}}|x|^b u(x)\,dx\,. $$

If $\lambda ^*$ minimises $M(\lambda )$, then $M'(\lambda ^*)=0$, where $M'(\lambda ^*)$ is given by

$$ Q\left( \frac{1}{\alpha }-1\right) (\lambda ^{*})^{Q(\frac{1}{\alpha }-1)-1}+\left[ Q\left( \frac{1}{\alpha }-1\right) -b\right] (\lambda ^*)^{Q(\frac{1}{\alpha }-1)-b-1}\int _{\mathbb {G}}|x|^b u(x)dx\,. $$

One can easily check that for

$$\begin{aligned} \lambda ^*=\left\{ \left( \frac{\alpha b-Q(1-\alpha )}{Q(1-\alpha )}\right) \int _{\mathbb {G}}|x|^b u(x)dx\right\} ^\frac{1}{b} \end{aligned}$$

we have $M'(\lambda ^*)=0$, and we we can compute

$$\begin{aligned} \begin{aligned} M(\lambda ^*)&\ = (\lambda ^{*})^{Q\left( \frac{1}{\alpha }-1\right) }\left[ 1+ (\lambda ^{*})^{-b}\int _{\mathbb {G}}|x|^b u(x)\,dx\right] \\  &= \left\{ \left( \frac{\alpha b-Q(1-\alpha )}{Q(1-\alpha )}\right) \int _{\mathbb {G}}|x|^b u(x)dx\right\} ^{\frac{Q}{b}\left( \frac{1}{\alpha }-1 \right) } \\  &\times \left[ 1+\frac{Q(1-\alpha )}{\alpha b-Q(1-\alpha )}\left( \int _{\mathbb {G}}|x|^bu(x)\,dx \right) ^{-1}\int _{\mathbb {G}}|x|^b u(x)\,dx \right] \\  &=\frac{\alpha b}{\alpha b-Q(1-\alpha )}\left\{ \left( \frac{\alpha b-Q(1-\alpha )}{Q(1-\alpha )}\right) \int _{\mathbb {G}}|x|^b u(x)dx\right\} ^{\frac{Q}{b}(\frac{1}{\alpha }-1)}\,. \end{aligned} \end{aligned}$$

Thus, inequality (18) for $\lambda =\lambda ^*$ becomes

19 $$\begin{aligned} \begin{aligned} \log \left( \frac{C_{1}\Vert u\Vert ^{\frac{1}{1-\alpha }}_{L^{\alpha }(\mathbb {G})}}{\Vert \phi _1\Vert _{L^{\alpha }(\mathbb {G})}}\right)&\ \le \frac{1}{(1-\alpha )}\log \bigg [\left( \frac{\alpha b}{\alpha b-Q(1-\alpha )}\right) \times \\  &\ \left\{ \left( \frac{\alpha b-Q(1-\alpha )}{Q(1-\alpha )}\right) \int _{\mathbb {G}}|x|^b u(x)dx\right\} ^{\frac{Q}{b}(\frac{1}{\alpha }-1)}\bigg ]\,. \end{aligned} \end{aligned}$$

Now, in order to rewrite the equation (19) in the form of (11), observe that (19) can equivalently be written as

$$ \begin{aligned} \log \left( \Vert u\Vert _{L^{\alpha }(\mathbb {G})}^{\frac{1}{1-\alpha }}\right)&\ \le \log \left( \frac{\Vert \phi _1\Vert _{L^{\alpha }(\mathbb {G})}}{C_1}\right) \\&\ +\frac{1}{1-\alpha }\log \left[ \frac{\alpha b}{\alpha b -Q(1-\alpha )} \left\{ \left( \frac{\alpha b -Q(1-\alpha )}{Q(1-\alpha )}\right) I\right\} ^{\frac{Q}{b}\left( \frac{1}{\alpha }-1 \right) }\right] \,, \end{aligned} $$

where we have denoted $I:=\int _{\mathbb {G}}|x|^b u(x)\,dx$. Now, multiplying the latter by $\alpha$ we obtain

$$ \begin{aligned} \log \left( \int _{\mathbb {G}}|u(x)|^{\alpha }\,dx\right) ^{\frac{1}{1-\alpha }}&\ \le \log \left( \frac{\Vert \phi _1\Vert _{L^{\alpha }(\mathbb {G})}^{\alpha }}{C_1^\alpha }\right) \\ +&\ \frac{\alpha }{1-\alpha }\log \left[ \frac{\alpha b}{\alpha b -Q(1-\alpha )} \left\{ \left( \frac{\alpha b -Q(1-\alpha )}{Q(1-\alpha )}\right) I\right\} ^{\frac{Q}{b}\left( \frac{1}{\alpha }-1 \right) }\right] \,. \end{aligned} $$

Observe that the first term of the RHS in the above inequality can be written as

20 $$\begin{aligned} \frac{Q}{b}\log \left( \frac{\Vert \phi _1\Vert _{L^{\alpha }(\mathbb {G})}^\alpha }{C_1^{\alpha }}\right) ^{\frac{b}{Q}}\,, \end{aligned}$$

while the second term of the RHS can be written as

21 $$\begin{aligned} \frac{Q}{b} \log \left( \left( \frac{\alpha b}{\alpha b-Q(1-\alpha )}\right) ^{\frac{\alpha b}{Q(1-\alpha )}} \left( \frac{\alpha b-Q(1-\alpha )}{Q(1-\alpha )} \right) I\right) \,. \end{aligned}$$

Now, using (A11) we see that the (20) can be written as

22 $$\begin{aligned} \frac{Q}{b}\log \left( \frac{\Vert \phi _1\Vert _{L^{\alpha }(\mathbb {G})}^\alpha }{C_1^{\alpha }}\right) ^{\frac{b}{Q}}=\frac{Q}{b} \log C_{1}^{-\frac{b}{Q}} \left( \frac{\alpha b}{\alpha b-Q(1-\alpha )}\right) ^{\frac{b}{Q}}\,. \end{aligned}$$

Now a combination of (22) together with (21) gives that

$$ \begin{aligned} A_{\alpha ,Q,b}&\ = C_{1}^{-\frac{b}{Q}} \left( \frac{\alpha b}{\alpha b-Q(1-\alpha )}\right) ^{\frac{b}{Q}} \frac{\alpha b}{\alpha b-Q(1-\alpha )}^{\frac{\alpha b}{Q(1-\alpha )}} \left( \frac{\alpha b-Q(1-\alpha )}{Q(1-\alpha )} \right) \\&= C_{1}^{-\frac{b}{Q}} \left( \frac{\alpha b}{\alpha b-Q(1-\alpha )}\right) ^{\frac{b}{Q(1-\alpha )}} \left( \frac{\alpha b-Q(1-\alpha )}{Q(1-\alpha )} \right) \,. \end{aligned} $$

We now treat the case where $\alpha >1$. We consider the auxiliary function $\phi _2$ given by

$$ \phi _2(x)=C_2(1-|x|^b)_{+}^{\frac{1}{\alpha -1}}\,, $$

with $C_2$ given in (A13). Using Jensen’s inequality for the concave function $G(x)=-x\log x$ and with the probability measure $\frac{u(x)^\alpha }{\Vert u\Vert _{L^{\alpha }(\mathbb {G})}^{\alpha }}dx$ we estimate

$$\begin{aligned} \int _{\mathbb {G}}u(x)\phi _2(x)^{\alpha -1}\log \frac{\phi _2(x)^{\alpha -1}}{u(x)^{\alpha -1}}\,dx= &   \int _{\mathbb {G}} u(x)^{\alpha }\frac{\phi _2(x)^{\alpha -1}}{u(x)^{\alpha -1}} \log \frac{\phi _2(x)^{\alpha -1}}{u(x)^{\alpha -1}}dx \nonumber \\= &   -\int _{\mathbb {G}} u(x)^\alpha \,G\left( \frac{\phi _2(x)^{\alpha -1}}{u(x)^{\alpha -1}}\right) \,dx \\= &   -\Vert u\Vert ^{\alpha }_{L^{\alpha }(\mathbb {G})}\int _{\mathbb {G}} \frac{u(x)^\alpha }{\Vert u\Vert _{L^{\alpha }(\mathbb {G})}^\alpha }G\left( \frac{\phi _2(x)^{\alpha -1}}{u(x)^{\alpha -1}}\right) dx\\\ge &   -\Vert u\Vert ^{\alpha }_{L^{\alpha }(\mathbb {G})} G\left( \frac{1}{\Vert u\Vert _{L^{\alpha }(\mathbb {G})}^{\alpha }}\int _{\mathbb {G}}u(x)\,\phi _2(x)^{\alpha -1}dx\right) . \end{aligned}$$

Now, if we denote by $K=K(u, \phi _2,\alpha )$ the quantity

23 $$\begin{aligned} K:=\int _{\mathbb {G}}u(x)\phi _2(x)^{\alpha -1}\,dx=\int _{\mathbb {G}}u(x)(C_2(1-|x|^b)_{+}^{\frac{1}{\alpha -1}})^{\alpha -1}\,dx < \infty \,, \end{aligned}$$

then, by the above computations, and the definition of the function *G*, we get

24 $$\begin{aligned} \int _{\mathbb {G}}u(x)\phi _2(x)^{\alpha -1}\log \frac{\phi _2(x)^{\alpha -1}}{u(x)^{\alpha -1}}\,dx \ge K \log \frac{K}{\Vert u\Vert ^{\alpha }_{L^{\alpha }(\mathbb {G})}}\,. \end{aligned}$$

On the other hand, by the properties of the logarithm we have

25 $$\begin{aligned} \int _{\mathbb {G}}u(x)\phi _2(x)^{\alpha -1}\log \frac{\phi _2(x)^{\alpha -1}}{u(x)^{\alpha -1}}\,dx= (\alpha -1)K \int _{\mathbb {G}} \frac{u(x)\phi _2(x)^{\alpha -1}}{K} \log \frac{\phi _2(x)}{u(x)}\,dx\,. \end{aligned}$$

Another application of Jensen’s inequality to the right-hand side of (25) yields

26 $$\begin{aligned} \int _{\mathbb {G}}K^{-1}u(x)\phi _2(x)^{\alpha -1}\log \frac{\phi _2(x)^{\alpha -1}}{u(x)^{\alpha -1}}\,dx \le (\alpha -1)\log (K^{-1}\Vert \phi _2\Vert _{L^{\alpha }(\mathbb {G})}^{\alpha })\,, \end{aligned}$$

where $\Vert \phi _2\Vert _{L^{\alpha }(\mathbb {G})}^{\alpha }$ has been computed in the Appendix, see (A14), as

$$ \Vert \phi _2\Vert _{L^{\alpha }(\mathbb {G})}^{\alpha }= \Vert \phi _2\Vert _{L^{\alpha }(\mathbb {G})}^{\alpha }=C_{2}^{\alpha -1}\frac{\alpha b }{b \alpha +Q(\alpha -1)}\,. $$

Hence by (25) and (26) we get

27 $$\begin{aligned} \int _{\mathbb {G}}u(x)\phi _2(x)^{\alpha -1}\log \frac{\phi _2(x)^{\alpha -1}}{u(x)^{\alpha -1}}\,dx \le (\alpha -1)K \log \frac{\Vert \phi _2\Vert _{L^{\alpha }(\mathbb {G})}^{\alpha }}{K}\,. \end{aligned}$$

A combination of (24) and (27) gives

28 $$\begin{aligned} \log \frac{K}{\Vert u\Vert _{L^{\alpha }(\mathbb {G})}^{\alpha }} \le (\alpha -1) \log \frac{\Vert \phi _2\Vert _{L^{\alpha }(\mathbb {G})}^{\alpha }}{K}\,, \end{aligned}$$

or equivalently

$$ \alpha \log \frac{K}{\Vert \phi _2\Vert _{L^{\alpha }(\mathbb {G})}^{\alpha -1}\Vert u\Vert _{L^{\alpha }(\mathbb {G})}}\le 0\,, $$

and since $\log x \le 0$ for $x\le 1$, the latter implies that

29 $$\begin{aligned} K \le \Vert \phi _2\Vert _{L^{\alpha }(\mathbb {G})}^{\alpha -1}\Vert u\Vert _{L^{\alpha }(\mathbb {G})}\,. \end{aligned}$$

Now, by (23), and since clearly $(1-|x|^b)_{+}\ge (1-|x|^b)$, we have

30 $$\begin{aligned} C^{\alpha -1}_{2} \int _{\mathbb {G}} (1-|x|^b)u(x)\,dx \le K. \end{aligned}$$

A combination of (29) and (30) gives

$$ C^{\alpha -1}_{2} \int _{\mathbb {G}} (1-|x|^b)u(x)\,dx \le \Vert \phi _2\Vert _{L^{\alpha }(\mathbb {G})}^{\alpha -1}\Vert u\Vert _{L^{\alpha }(\mathbb {G})}\,, $$

or, using the fact that $\Vert u\Vert _{L^1(\mathbb {G})}=1$, the latter can be simplified as follows

31 $$\begin{aligned} C_{2}^{\alpha -1}\le C_{2}^{\alpha -1}\int _{\mathbb {G}}|x|^bu(x)\,dx+\Vert \phi _2\Vert _{L^{\alpha }(\mathbb {G})}^{\alpha -1}\Vert u\Vert _{L^{\alpha }(\mathbb {G})}\,. \end{aligned}$$

For $\tilde{u}$ as before, the right-hand side of (31) becomes

$$ \lambda ^{-b}C_{2}^{\alpha -1}\int _{\mathbb {G}}|x|^b u(x)\,dx+\lambda ^{Q\left( 1-\frac{1}{\alpha } \right) }\Vert \phi _2\Vert _{L^{\alpha }(\mathbb {G})}^{\alpha -1}\Vert u\Vert _{L^{\alpha }(\mathbb {G})}\,. $$

Therefore, for any $\lambda >0$ the following inequality holds true

32 $$\begin{aligned} C_{2}^{\alpha -1}\le \lambda ^{-b}C_{2}^{\alpha -1}\int _{\mathbb {G}}|x|^b u(x)\,dx+\lambda ^{Q\left( 1-\frac{1}{\alpha } \right) }\Vert \phi _2\Vert _{L^{\alpha }(\mathbb {G})}^{\alpha -1}\Vert u\Vert _{L^{\alpha }(\mathbb {G})}:=N(\lambda )\,. \end{aligned}$$

Our next aim is, as we did above, to minimise the quantity $N(\lambda )$ over $\lambda$. Now, if we set $I=\int _{\mathbb {G}}|x|^bu(x)\,dx$, then we can write

$$ N(\lambda )= \lambda ^{-b}C_{2}^{\alpha -1}I+\lambda ^{Q\left( 1-\frac{1}{\alpha } \right) }\Vert \phi _2\Vert _{L^{\alpha }(\mathbb {G})}^{\alpha -1}\Vert u\Vert _{L^{\alpha }(\mathbb {G})}\,, $$

and we have

$$ N'(\lambda )= (-b)\lambda ^{-b-1}C_{2}^{\alpha -1}I+Q\left( 1-\frac{1}{\alpha } \right) \lambda ^{Q\left( 1-\frac{1}{\alpha } \right) -1}\Vert \phi _2\Vert _{L^{\alpha }(\mathbb {G})}^{\alpha -1}\Vert u\Vert _{L^{\alpha }(\mathbb {G})}\,. $$

It is then not difficult to check that $$N'(\lambda ^{**})=0$$, for

$$ \lambda ^{**}= \left( \frac{b \alpha C_{2}^{\alpha -1}I}{\Vert \phi _2\Vert _{L^{\alpha }(\mathbb {G})}^{\alpha -1}Q(\alpha -1)\Vert u\Vert _{L^{\alpha }(\mathbb {G})}} \right) ^{\frac{\alpha }{b \alpha +Q(\alpha -1)}}\,. $$

A direct substitution then gives

33 $$\begin{aligned} \begin{aligned} N(\lambda ^{**})&\ = \left( \frac{b \alpha C_{2}^{\alpha -1}I}{\Vert \phi _2\Vert _{L^{\alpha }(\mathbb {G})}^{\alpha -1}Q(\alpha -1)\Vert u\Vert _{L^{\alpha }(\mathbb {G})}} \right) ^{\frac{-b\alpha }{b \alpha +Q(\alpha -1)}}C_{2}^{\alpha -1}I \\  &+ \left( \frac{b \alpha C_{2}^{\alpha -1}I}{\Vert \phi _2\Vert _{L^{\alpha }(\mathbb {G})}^{\alpha -1}Q(\alpha -1)\Vert u\Vert _{L^{\alpha }(\mathbb {G})}} \right) ^{\frac{\alpha Q}{b \alpha +Q(\alpha -1)}\left( 1-\frac{1}{\alpha } \right) }\Vert \phi _2\Vert _{L^{\alpha }(\mathbb {G})}^{\alpha -1}\Vert u\Vert _{L^{\alpha }(\mathbb {G})}\,. \end{aligned} \end{aligned}$$

Observe that since

$$ -\left( \frac{-b\alpha }{b \alpha +Q(\alpha -1)} \right) =-\left[ \frac{\alpha Q}{b \alpha +Q(\alpha -1)}\left( 1-\frac{1}{\alpha } \right) \right] +1=\frac{\alpha b}{Q(\alpha -1)+\alpha b}\,, $$

and

$$ \left[ \frac{-b\alpha }{b \alpha +Q(\alpha -1)} \right] +1=\frac{\alpha Q}{b \alpha +Q(\alpha -1)}\left( 1-\frac{1}{\alpha }\right) =\frac{Q(\alpha -1)}{Q(\alpha -1)+\alpha b} $$

the quantities $I^{\frac{Q(\alpha -1)}{Q(\alpha -1)+\alpha b}}$ and $\Vert u\Vert _{L^{\alpha (\mathbb {G})}}^{\frac{\alpha b}{Q(\alpha -1)+\alpha b}}$ are common multiplies of the summands in (33), and we can write

34 $$\begin{aligned} N(\lambda ^{**})=S I^{\frac{Q(\alpha -1)}{Q(\alpha -1)+\alpha b}} \Vert u\Vert _{L^{\alpha (\mathbb {G})}}^{\frac{\alpha b}{Q(\alpha -1)+\alpha b}} \end{aligned}$$

where we have denoted by $S=S(\alpha ,b,Q)$ the following quantity

35 $$\begin{aligned} S= &   \left( \frac{\alpha b}{Q\left( \alpha -1\right) }\right) ^{\frac{-\alpha b}{Q(\alpha -1)+\alpha b}} C^{\frac{{Q(\alpha -1)^2}}{Q(\alpha -1)+\alpha b}}_{2}\,\Vert \phi _2\Vert _{L^{\alpha }(\mathbb {G})}^{\frac{\alpha b(\alpha -1)}{Q(\alpha -1)+\alpha b}}\left( \frac{\alpha b+Q(\alpha -1)}{Q(\alpha -1)}\right) \nonumber \\= &   \left( \frac{\alpha b+Q(\alpha -1)}{\alpha b}\right) ^{\frac{b+Q(\alpha -1)}{\alpha b+Q(\alpha -1)}}\left( \frac{\alpha b}{Q\left( \alpha -1\right) }\right) ^\frac{Q(\alpha -1)}{\alpha b+Q(\alpha -1)}C^{\frac{{(Q+b)(\alpha -1)^2}}{Q(\alpha -1)+\alpha b}}_{2}, \end{aligned}$$

and for the last equality we have used the expression (A7) for $\Vert \phi _2\Vert ^{\alpha }_{L^{\alpha }(\mathbb {G})}$.

Hence by (32) and (34) we get

$$\begin{aligned}&1\le SC^{1-\alpha }_{2} \left( \int _{\mathbb {G}}|x|^bu(x) dx\right) ^{\frac{Q(\alpha -1)}{Q(\alpha -1)+\alpha b}} \Vert u\Vert _{L^{\alpha }(\mathbb {G})}^{\frac{\alpha b}{Q(\alpha -1)+\alpha b}}\,, \end{aligned}$$

where we have substituted the expression for *I*, and the latter implies that

$$\begin{aligned}&- \log \left( \Vert u\Vert _{L^{\alpha }(\mathbb {G})}^{\frac{\alpha b}{Q(\alpha -1)+\alpha b}} \right) \le \log \left[ SC^{1-\alpha }_{2} \left( \int _{\mathbb {G}}|x|^bu(x) dx\right) ^{\frac{Q(\alpha -1)}{Q(\alpha -1)+\alpha b}}\right] \,. \end{aligned}$$

Hence we get

$$\begin{aligned}&-\frac{b}{Q(\alpha -1)+\alpha b}\log \int _{\mathbb {G}}u(x)^{\alpha }\,dx\\  &\quad \le \ \frac{Q(\alpha -1)}{Q(\alpha -1)+\alpha b} \log \left( [SC^{1-\alpha }_{2}]^{\frac{Q(\alpha -1)+\alpha b}{Q(\alpha -1)}} \int _{\mathbb {G}}|x|^bu(x)\,dx\right) \,, \end{aligned}$$

or after simplifications

36 $$\begin{aligned} \frac{1}{1-\alpha } \log \int _\mathbb {G}u(x)^\alpha \, dx \le \frac{Q}{b} \log \Bigg ( [C^{1-\alpha }_{2} S]^{\frac{b\alpha }{Q(\alpha -1)}+1} \int _\mathbb {G}|x|^b u(x) dx \Bigg ), \end{aligned}$$

and we have obtained (11) for $\alpha >1$ with $A_{\alpha ,Q,b}$ given by

37 $$\begin{aligned} \begin{aligned} A_{\alpha ,Q,b}&\ = [C^{1-\alpha }_{2} S]^{\frac{b\alpha }{Q(\alpha -1)}+1} \\  &= \left[ \left( \frac{\alpha b+Q(\alpha -1)}{\alpha b}\right) ^{\frac{b+Q(\alpha -1)}{\alpha b+Q(\alpha -1)}}\left( \frac{\alpha b}{Q\left( \alpha -1\right) }\right) ^\frac{Q(\alpha -1)}{\alpha b+Q(\alpha -1)} \right] ^{\frac{b\alpha }{Q(\alpha -1)}+1} \\  &\times C_{2}^{\left\{ (1-\alpha )+\frac{(Q+b)(\alpha -1)^2}{Q(\alpha -1)+\alpha b}\right\} \left\{ \frac{b\alpha }{Q(\alpha -1)}+1\right\} } \\  &=\frac{\alpha b}{Q(\alpha -1)}\left( \frac{\alpha b +Q(\alpha -1)}{\alpha b} \right) ^{\frac{Q(\alpha -1)+b}{Q(\alpha -1)}}\left( \frac{b}{|\mathfrak {S}|}\frac{\Gamma \left( \frac{\alpha }{\alpha -1}+\frac{Q}{b}\right) }{\Gamma (\frac{\alpha }{\alpha -1})\Gamma (\frac{Q}{b})} \right) ^{-\frac{b}{Q}}\,, \end{aligned} \end{aligned}$$

where we have used the expressions for *S* and $C_2$ given in (35) and (A13), respectively. This completes the proof of the Theorem 3.1.

The proving procedure that we followed in Theorem 3.1 show that the inequality (11) is sharp. Indeed we have the following result:

### Corollary 3.2

The constant $A_{\alpha ,Q,b}$ in the inequality (11) is sharp. In particular, when $\alpha \in (0,1)$, the inequality (11) holds true as as equality for (up to $L^1(\mathbb {G})$-scaling) $u=\phi _1$ where $\phi _1(x)=C_1(1+|x|^b)^{\frac{1}{\alpha -1}}$ and $C_1$ is given by (A6). Similarly, when $\alpha >1$, the inequality (11) holds true as an equality for (up to $L^{1}(\mathbb {G})$-scaling) $u=\phi _2$, where $\phi _2(x)=C_2 (1+|x|^b)^{\frac{1}{\alpha -1}}_{+}$, with $C_2$ given in (A13).

### Remark 3.3

Using simple mathematical arguments, it is easy to check that the left-hand side of the inequality (11), i.e., of the Rényi entropy, when $\alpha \rightarrow 1$ approximates the Shannon entropy, i.e., the quantity

$$ -\int _{\mathbb {G}}u(x) \log u(x)\,dx\,. $$

In [3] the authors proved the optimal Shannon inequality on homogeneous Lie groups that reads as follows

38 $$\begin{aligned} -\int _{\mathbb {G}}u(x) \log u(x)\,dx \le \frac{Q}{2} \log \left( C_\mathbb {G}\int _\mathbb {G}|x|^2 u(x)\,dx \right) \,, \end{aligned}$$

where the best constant $C_\mathbb {G}$ is given by

39 $$\begin{aligned} C_\mathbb {G}=\frac{2e}{Q}\left( \frac{|\mathfrak {S}|\Gamma \left( \frac{Q}{2} \right) }{2}\right) ^{\frac{2}{Q}}\,, \end{aligned}$$

and we have $C_{\mathbb {R}^n}=\frac{2e\pi }{n}$, as expected, since the constant $C_{\mathbb {G}}$ is optimal.

In the next result, by taking the limit of the Rényi entropy, we also obtain the so-called Shannon inequality on homogeneous Lie groups. As expected, the sharpness of the constant in the inequality (11) implies an optimal bound for the Shannon entropy in the aforesaid setting which is exactly the quantity on the right-hand side of (38).

### Theorem 3.4

Let $\mathbb {G}$ be a homogeneous Lie group of homogeneous dimension *Q*, and suppose that $|\cdot |$ is a homogeneous quasi-norm on $\mathbb {G}$. Then Shannon inequality reads as follows

40 $$\begin{aligned} -\int _{\mathbb {G}}u(x) \log u(x)\,dx \le \frac{Q}{2} \log \left( C_\mathbb {G}\int _\mathbb {G}|x|^2 u(x)\,dx \right) \,, \end{aligned}$$

where

$$ C_{\mathbb {G}}= \frac{2e}{Q}\left( \frac{|\mathfrak {S}|\Gamma \left( \frac{Q}{2} \right) }{2}\right) ^{\frac{2}{Q}}\,. $$

### Proof

We first consider the case where $\alpha >1$. By Remark 3.3 it is enough to show that $\lim _{\alpha \rightarrow 1}A_{\alpha ,Q,2}=C_{\mathbb {G}}$, where $A_{\alpha ,Q,2}$ is given by Theorem 3.1 for $\beta =2$, and $\alpha >1$. If we set $c=\frac{\alpha }{\alpha -1}$, then $c \rightarrow \infty$, when $\alpha \rightarrow 1$, and we have

$$ A_{c(\alpha ),Q,2}=\frac{2c}{Q}\left( 1+\frac{Q}{2c} \right) ^{\frac{2c}{\alpha Q}+1} \left( \frac{2}{|\mathfrak {S}|}\frac{\Gamma \left( c+\frac{Q}{2}\right) }{\Gamma \left( c \right) \Gamma \left( \frac{Q}{2} \right) }\, \right) ^{-\frac{2}{Q}}\,. $$

To calculate the quantity $\left( \frac{\Gamma \left( c \right) }{\Gamma \left( c+\frac{Q}{2} \right) }\, \right) ^{\frac{2}{Q}}$, when $c \gg 1$, we will use the Stirling approximation formula:

41 $$\begin{aligned} \Gamma (x)\simeq \sqrt{2 \pi } e^{-x}x^{x-1/2}\,,\quad \text {when} \quad x \gg 1\,. \end{aligned}$$

We have

$$\begin{aligned} \left( \frac{\Gamma \left( c \right) }{\Gamma \left( c+\frac{Q}{2} \right) }\, \right) ^{\frac{2}{Q}}\simeq &   \left[ \frac{\sqrt{2 \pi }e^{-c}c^{c-1/2}}{\sqrt{2 \pi } e^{-c-Q/2}(c+Q/2)^{c+Q/2-1/2}}\right] ^{\frac{2}{Q}}\\= &   e c^{\frac{2c}{Q}-\frac{1}{Q}} (c+Q/2)^{-1-\frac{2c}{Q}+\frac{1}{Q}}. \end{aligned}$$

Since

$$ \lim _{c \rightarrow \infty , \alpha \rightarrow 1}\left( 1+\frac{Q}{2c} \right) ^{\frac{2c}{\alpha Q}+1} =1\,, $$

we have

$$\begin{aligned} \lim _{c \rightarrow \infty ,\alpha \rightarrow 1} A_{c(\alpha ),Q,2}= &   \frac{2e}{Q}\left( \frac{2}{|\mathfrak {S}|}\Gamma \left( \frac{Q}{2} \right) ^{-1}\right) ^{-\frac{2}{Q}} \lim _{c \rightarrow \infty } c c^{\frac{2c}{Q}-\frac{1}{Q}} (c+Q/2)^{-1-\frac{2c}{Q}+\frac{1}{Q}}\\= &   \frac{2e}{Q}\left( \frac{2}{|\mathfrak {S}|}\Gamma \left( \frac{Q}{2} \right) ^{-1}\right) ^{-\frac{2}{Q}}\\= &   \frac{2e}{Q}\left( \frac{|\mathfrak {S}|}{2}\Gamma \left( \frac{Q}{2} \right) \right) ^{\frac{2}{Q}} , \end{aligned}$$

and this completes the proof for the case where $\alpha >1$. As expected, the same constant appears when $0<\alpha <1$; Indeed, for $b=2$ in (11) we have

42 $$\begin{aligned} A_{\alpha ,Q,2} = \Bigg ( \frac{2}{|\mathfrak {S}|}\, \frac{\Gamma \!\left( \tfrac{1}{1-\alpha }\right) }{\Gamma \!\left( \tfrac{1}{1-\alpha }-\tfrac{Q}{2}\right) \, \Gamma \!\left( \tfrac{Q}{2}\right) } \Bigg )^{-\frac{2}{Q}} \Bigg ( \frac{2\alpha }{2\alpha - Q(1-\alpha )} \Bigg )^{\frac{2}{Q(1-\alpha )}} \Bigg ( \frac{2\alpha - Q(1-\alpha )}{Q(1-\alpha )} \Bigg ). \end{aligned}$$

Set

$$ d:=\frac{1}{1-\alpha },\qquad \alpha =\frac{d-1}{d} \qquad \text {so that } \quad d\rightarrow \infty \ \text { as } \ \alpha \rightarrow 1^{-}. $$

Then the first factor in (42) becomes

$$ \Bigg ( \frac{2}{|\mathfrak {S}|} \frac{\Gamma (d)}{\Gamma \!\left( d-\frac{Q}{2}\right) \Gamma (\frac{Q}{2})} \Bigg )^{-\frac{2}{Q}}. $$

By the Stirling formula (41) we have

$$ \frac{\Gamma (d)}{\Gamma (d-\frac{Q}{2})} \sim d^{\frac{Q}{2}}, $$

and so

43 $$\begin{aligned} \Bigg ( \frac{2}{|\mathfrak {S}|} \frac{\Gamma (d)}{\Gamma \!\left( d-\frac{Q}{2}\right) \Gamma (\frac{Q}{2})} \Bigg )^{-\frac{2}{Q}} \sim \Bigg ( \frac{2}{|\mathfrak {S}|\Gamma (\frac{Q}{2})} \Bigg )^{-\frac{2}{Q}} \, d^{-1}. \end{aligned}$$

Next, observe that

$$ \frac{2\alpha }{2\alpha - Q(1-\alpha )} = \frac{2 - \frac{2}{d}}{\,2 - \frac{Q+2}{d}\,} = \frac{1 - \frac{1}{d}}{1 - \frac{Q+2}{2d}}=\Big (1 - \frac{1}{d}\Big ) \Big (1 - \frac{(Q+2)/2}{d}\Big )^{-1}\,. $$

Hence, using that

$$ \Big (1 - \frac{(Q+2)/2}{d}\Big )^{-1} = 1 + \frac{(Q+2)/2}{d} + O(d^{-2}). $$

we conclude that

$$ \frac{2\alpha }{2\alpha - Q(1-\alpha )} = 1 + \frac{Q}{2d} + O(d^{-2}), \qquad d\rightarrow \infty . $$

and so also that

44 $$\begin{aligned} \Bigg ( \frac{2\alpha }{2\alpha - Q(1-\alpha )} \Bigg )^{\frac{2d}{Q}} \longrightarrow e \qquad (d\rightarrow \infty ). \end{aligned}$$

For the last factor we have

45 $$\begin{aligned} \frac{2\alpha - Q(1-\alpha )}{Q(1-\alpha )} = \frac{2\alpha }{Q}(1-\alpha )^{-1} + O(1) = \frac{2\alpha }{Q}d + O(1). \end{aligned}$$

Combining (43), (44), and (45), we obtain

$$ A_{\alpha ,Q,2} \sim \Bigg ( \frac{2}{|\mathfrak {S}|\Gamma (\frac{Q}{2})} \Bigg )^{-\frac{2}{Q}} d^{-1} \,\cdot \, e \,\cdot \, \frac{2}{Q}d = \frac{2e}{Q} \Bigg (\frac{|\mathfrak {S}|\Gamma (\frac{Q}{2})}{2}\Bigg )^{\frac{2}{Q}}. $$

Summarising, we have shown that

$$ \displaystyle \lim _{\alpha \rightarrow 1^{-}} A_{\alpha ,Q,2} = C_\mathbb {G}= \displaystyle \lim _{\alpha \rightarrow 1^{+}} A_{\alpha ,Q,2}\,, $$

completing the proof of (40) also in the case $0<\alpha <1$. $\square$

The next result applies to a subclass of graded Lie groups when $V_1$ as in (8) generates the whole of the Lie algebra. Precisely, if $\mathbb {G}$ is a graded Lie group with Lie algebra $\mathfrak {g}$ that admits a gradation of the form (8) and where the elements $\{X_1,\cdots ,X_k\}$, that are such that $V_1=\text {span}\{X_1,\cdots ,X_k\}$, generate after iterated commutators the whole of $\mathfrak {g}$.

In the case of stratified Lie groups the elliptic Laplace operator $\Delta$ on $\mathbb {R}^n$ is replaced by the (positive) hypoelliptic operator $\Delta _{\text {sub}}$, that is commonly called a sub-Laplacian on $\mathbb {G}$, defined via

$$ \Delta _{\text {sub}}=-\sum _{i=1}^{k}X_{i}^{2}\,, $$

and we have $\Delta _{\text {sub}}=-\nabla _{H}^{*}\nabla _{H}$, where $\nabla _{H}$ is called the horizontal gradient on $\mathbb {G}$; namely $\nabla _{H}$ is the vector valued operator acting on *f* via

$$ \nabla _{H}f=(X_1 f,\cdots , X_k f)\,. $$

Let us recall a version of the logarithmic Sobolev inequality in the setting of stratified groups, as appeared in [4, Corollary 7.1]

### Proposition 3.5

Let $\mathbb {G}$ be a stratified Lie group with homogeneous dimension *Q*. Then, the following log-Sobolev inequality is satisfied

46 $$\begin{aligned} \int _{\mathbb {G}}|f|^2 \log |f|\,dx \le \frac{Q}{4}\log \left( A \int _{\mathbb {G}}|\nabla _{H}f|^2\,dx \right) \,, \end{aligned}$$

for every *f* such that $\Vert f\Vert _{L^2(\mathbb {G})}=1$, for some constant *A* with a formula that depends on the group $\mathbb {G}$, and in particular it depends also on its homogeneous dimension *Q*.

Let us point out that the constant *A* is related to the Gagliardo-Nirenberg inequalities in our setting, and cannot be yet explicitly computed in the general setting of a stratified Lie group. However, in the particular case of the Heisenberg group we have $A=\frac{(n!)^{\frac{1}{n+1}}}{\pi n^2}$, see [5, 11], and when $\mathbb {G}=\mathbb {R}^n$ we have $A=(\pi n^2-2\pi n)^{-1/2}\frac{\Gamma (n)}{\Gamma (n/2)}$, see [23], and in these cases *A* is optimal. To avoid technicalities we do not include the explicit formula for *A* here, and we refer the interested reader to [4, 9, 18].

### Theorem 3.6

Let $\mathbb {G}$ be a stratified Lie group with homogeneous dimension *Q*, and let $u \in L^1(\mathbb {G}) \cap L^2(\mathbb {G})$ be a nonnegative function such that $\Vert u\Vert _{L^1(\mathbb {G})}=1.$ Then it holds that

47 $$\begin{aligned} \int _{\mathbb {G}}u(x) \log u(x)\,dx \le \frac{Q}{2} \log \left( \frac{A}{4}\int _{\mathbb {G}}\frac{1}{u(x)}|\nabla _{H}u|^2\,dx \right) , \end{aligned}$$

where the constant *A* is the one appearing in (46).

### Proof

Let *u* be a function as in the hypothesis. The proof follows by inequality (46) for *u* such that $f=u^{\frac{1}{2}}$. $\square$

As it happens in the Euclidean setting, see [12], a combination of the logarithmic Sobolev inequality of the form (47) with the Shannon’s inequality (40) yields the Heisenberg uncertainty principle in the setting of stratified groups.

### Corollary 3.7

A version of the Heisenberg uncertainty principle in the setting of stratified groups reads as follows:

48 $$\begin{aligned} \left( \int _{\mathbb {G}}|x|^2 u(x)\,dx \right) \left( \int _{\mathbb {G}}\frac{1}{u(x)}|\nabla _{H}u|^2\,dx \right) \ge 4C_{\mathbb {G}}^{-1}A^{-1}\,, \end{aligned}$$

for all functions *u* as in the hypothesis of Theorem 3.6, where $C_\mathbb {G}$ and *A* are given in (39), (46), respectively.

### Proof

A combination of (47) and (38) gives

$$ -\frac{Q}{2} \log \left( \frac{A}{4} \int _{\mathbb {G}}\frac{1}{u(x)}|\nabla _{H}u|^2\,dx \right) \le \frac{Q}{2} \log \left( C_{\mathbb {G}} \int _{\mathbb {G}}|x|^2 u(x)\,dx \right) \,. $$

Therefore, we have

$$ -\log (C_{\mathbb {G}}\frac{A}{4}) \le \log \left[ \left( \int _{\mathbb {G}}\frac{1}{u(x)}|\nabla _{H}u|^2\,dx \right) \left( \int _{\mathbb {G}}|x|^2 u(x)\,dx\right) \right] \,, $$

and by the properties of the logarithm, we obtain

$$ \left( \int _{\mathbb {G}}|x|^2 u(x)\,dx \right) \left( \int _{\mathbb {G}}\frac{1}{u(x)}|\nabla _{H}u|^2\,dx \right) \ge 4A^{-1}C_{\mathbb {G}}^{-1}\,, $$

and the proof is complete. $\square$

## Appendix A

For the proof of Theorem 3.1 we used the auxiliary functions $\phi _1,\phi _2\in L^1(\mathbb {G})\cap L^1_b(\mathbb {G})$, given by

$$\begin{aligned} \phi _1(x)=C_{1}\left( 1+|x|^b\right) ^{\frac{1}{\alpha -1}},\quad \text {where}\quad b>Q\left( \frac{1}{\alpha }-1\right) , \,\alpha \in (0,1)\,, \end{aligned}$$

A1 $$\begin{aligned} \phi _2(x)=C_{2}\left( 1-|x|^b\right) _+^{\frac{1}{\alpha -1}},\quad \text {where}\quad b>0,\,\alpha >1\,, \end{aligned}$$

where the constants $C_1=C_1(\alpha ,b,Q)$ and $C_2=C_2(\alpha ,b,Q)$ that we are computing below are such that $\Vert \phi _i\Vert _{L^1(\mathbb {G})}=1$, for $i=1,2$. The computations that follow, is a homogeneous group adaptation of the analogous computation in the Euclidean setting developed in [22].

Using the polar decomposition for the function $\phi _1$ we can write

$$\begin{aligned} \begin{aligned} \int _{\mathbb {G}}\left( 1+|x|^b\right) ^{\frac{1}{\alpha -1}}dx&= \int _0^\infty \int _\mathfrak {S}\left( 1+r^b\right) ^{\frac{1}{\alpha -1}}r^{Q-1}dr\,d\sigma (y)\\  &=|\mathfrak {S}|\int _0^\infty \left( 1+r^b\right) ^{\frac{1}{\alpha -1}}r^{Q-1}dr\,. \end{aligned} \end{aligned}$$

If we set $1+r^b=t$, so that $r=(t-1)^\frac{1}{b}$, then we can further compute

A2 $$\begin{aligned} \begin{aligned} \int _{\mathbb {G}}\left( 1+|x|^b\right) ^{\frac{1}{\alpha -1}}dx&= |\mathfrak {S}|\int _1^\infty t^{\frac{1}{\alpha -1}}\frac{(t-1)^\frac{(Q-1)}{b}}{b(t-1)^{1-\frac{1}{b}}}dt \\  &= \frac{|\mathfrak {S}|}{b}\int _1^\infty t^{\frac{1}{\alpha -1}}(t-1)^{\frac{Q}{b}-1}dt\,. \end{aligned} \end{aligned}$$

To further compute (A2), let us first recall the definition of the Beta function

A3 $$\begin{aligned} B(x,y)=\int _{0}^{1}t^{x-1}(1-t)^{y-1}dt\,, \end{aligned}$$

where $x,y \in \mathbb {C}$, with $\text {Re}(x), \text {Re}(y)>0$. Alternatively, one can obtain the following representation of *B*

A4 $$\begin{aligned} B(x,y)=\int _1^\infty w^{-(x+y)}(w-1)^{y-1}dw\,. \end{aligned}$$

For $-(x+y)=\frac{1}{\alpha -1}$ and $y=\frac{Q}{b}$, we have $x=\frac{1}{1-\alpha }-\frac{Q}{b}$, so that with the use of (A4) and the property (see e.g. [22]) which relates the Beta function with the Gamma function

$$ B(x,y)=\frac{\Gamma (x)\Gamma (y)}{\Gamma (x+y)}\,,\quad x,y \notin \mathbb {Z}_{0}^{-}\,, $$

due to (A2) and (A4) we have

A5 $$\begin{aligned}&\int _{\mathbb {G}}\left( 1+|x|^b\right) ^{\frac{1}{\alpha -1}}dx =\frac{|\mathfrak {S}|}{b}\,B\left( \frac{1}{1-\alpha }-\frac{Q}{b},\frac{Q}{b}\right) \nonumber \\  &\quad \quad \quad \quad \quad \quad \quad =\frac{|\mathfrak {S}|}{b}\frac{\Gamma \left( \frac{1}{1-\alpha }-\frac{Q}{b}\right) \Gamma (\frac{Q}{b})}{\Gamma \left( \frac{1}{1-\alpha }\right) }. \end{aligned}$$

Now, the requirement $\Vert \phi _1\Vert _{L^1(\mathbb {G})}=1$, yields

A6 $$\begin{aligned} C_{1}= \frac{b}{|\mathfrak {S}|}\frac{\Gamma \left( \frac{1}{1-\alpha }\right) }{\Gamma \left( \frac{1}{1-\alpha }-\frac{Q}{b}\right) \Gamma (\frac{Q}{b})}\,. \end{aligned}$$

Next we compute the $L^{\alpha }(\mathbb {G})$-norm of $\phi _1$. To this end, let us first compute the following integral

$$\begin{aligned} \int _{\mathbb {G}}|x|^b \phi _1(x)\,dx= &   C_{1} |\mathfrak {S}|\int _0^\infty r^b(1+r^b)^{\frac{1}{\alpha -1}} r^{Q-1}\,dr \nonumber \\= &   C_{1} |\mathfrak {S}|\int _0^\infty (1+r^b)^\frac{1}{\alpha -1} r^{b+Q-1}\,dr \\= &   C_{1} \frac{|\mathfrak {S}}{b}|\int _1^\infty t^{\frac{1}{\alpha -1}} (t-1)^{\frac{Q+b}{b}-1}\,dt, \end{aligned}$$

where we have used the change of variables $(1+r^b)=t$ implying that $r=(t-1)^\frac{1}{b}$. Now, using (A4) the latter can be expressed in the following form

A7 $$\begin{aligned} \int _{\mathbb {G}}|x|^b \phi _1(x)\,dx= &   C_{1} \frac{|\mathfrak {S}|}{b}\,B\left( \frac{1}{1-\alpha }-\frac{Q}{b}-1,\frac{Q}{b}+1\right) \nonumber \\= &   C_{1} \frac{|\mathfrak {S}|}{b}\,\frac{\Gamma (\frac{1}{1-\alpha }-\frac{Q}{b}-1)\Gamma \left( \frac{Q}{b}+1\right) }{\Gamma (\frac{1}{1-\alpha })}. \end{aligned}$$

Recall the following property of the Gamma function

$$\begin{aligned} z\Gamma (z)=\Gamma (z+1)\,, \quad \text {for all} \quad z\in \mathbb {C}, z \notin -\mathbb {N}\,. \end{aligned}$$

We then have

A8 $$\begin{aligned} \Gamma \left( \frac{1}{1-\alpha }-\frac{Q}{b}-1\right) = \frac{\Gamma \left( \frac{1}{1-\alpha }-\frac{Q}{b}\right) }{\left( \frac{1}{1-\alpha }-\frac{Q}{b}-1\right) }, \end{aligned}$$

and

A9 $$\begin{aligned} \Gamma \left( \frac{Q}{b}+1\right) =\frac{Q}{b}\Gamma \left( \frac{Q}{b}\right) \,. \end{aligned}$$

Using (A8) and (A9) in (A7), we obtain

A10 $$\begin{aligned}  &   \int _{\mathbb {G}} |x|^b \phi _1(x)\,dx x = C_{1} \frac{|\mathfrak {S}|}{b}\,\frac{\frac{\Gamma \left( \frac{1}{1-\alpha }-\frac{Q}{b}\right) }{\left( \frac{1}{1-\alpha }-\frac{Q}{b}-1\right) }\left( \frac{Q}{b}\right) \Gamma \left( \frac{Q}{b}\right) }{\Gamma (\frac{1}{1-\alpha })}\nonumber \\= &   \frac{Q(1-\alpha )}{\alpha b-Q(1-\alpha )}, \end{aligned}$$

where for the last equality we have used the formula for $C_1$ given in (A6).

Finally using (A10) and the fact that $\Vert \phi _1\Vert _{L^1(\mathbb {G})}=1$, we get

A11 $$\begin{aligned} \Vert \phi _1\Vert ^{\alpha }_{L^{\alpha }(\mathbb {G})}= &   \int _{\mathbb {G}}C_{1}^{\alpha }(1+|x|^b)^{\frac{\alpha }{\alpha -1}}\,dx \nonumber \\= &   \int _{\mathbb {G}}C_{1}^{\alpha -1}C_{1}(1+|x|^b)(1+|x|^b)^{\frac{1}{\alpha -1}}\,dx \nonumber \\= &   C_{1}^{\alpha -1} \int _{\mathbb {G}} (1+|x|^b)\phi _{1}(x)\,dx \nonumber \\= &   C_{1}^{\alpha -1} \left\{ \int _{\mathbb {G}}\phi _1(x)\,dx + \int _{\mathbb {G}}|x|^b \phi _1(x) \right\} \nonumber \\= &   C_{1}^{\alpha -1} \left\{ 1+ \frac{Q(1-\alpha )}{\alpha b -Q(1-\alpha )} \right\} \nonumber \\= &   C_{1}^{\alpha -1} \frac{\alpha b }{\alpha b- Q(1-\alpha )}. \end{aligned}$$

Let us now perform a similar analysis for the function $\phi _2$ as in (A1) where $b>0$ and $\alpha >1$. As before, we use polar decomposition and we have

$$\begin{aligned} \int _{\mathbb {G}}(1-|x|^b)_{+}^{\frac{1}{\alpha -1}}\,dx= &   \int _{\mathfrak {S}}\int _{0}^{1}\left( 1-r^b\right) ^{\frac{1}{\alpha -1}} r^{Q-1}dr\,d\sigma (y) \\= &   |\mathfrak {S}| \int _{0}^{1}\left( 1-r^b\right) ^{\frac{1}{\alpha -1}} r^{Q-1}dr . \end{aligned}$$

For $(1-r^b)=t$ we get

A12 $$\begin{aligned} \int _{\mathbb {G}}(1-|x|^b)_{+}^{\frac{1}{\alpha -1}}\,dx= &   |\mathfrak {S}|\int _0^1 t^{\frac{1}{\alpha -1}}\left( 1-t\right) ^{\frac{Q-1}{b}}\frac{1}{b}\left( 1-t\right) ^{\frac{1}{b}-1} dt \nonumber \\= &   \frac{|\mathfrak {S}|}{b}\int _0^1 t^{\frac{1}{\alpha -1}}\left( 1-t\right) ^{\frac{Q}{b}-1}\,dt. \end{aligned}$$

Arguing as before and using the definition of the Beta function as in (A3) for $x=\frac{\alpha }{\alpha -1}$ and $y=\frac{Q}{b}$, the condition $\Vert \phi _2\Vert _1=1$ yields

$$ \int _{\mathbb {G}}\phi _2(x)\,dx= \frac{C_{2}}{b}\,|\mathfrak {S}|\frac{\Gamma (\frac{\alpha }{\alpha -1})\Gamma (\frac{Q}{b})}{\Gamma \left( \frac{\alpha }{\alpha -1}+\frac{Q}{b}\right) }=1\,, $$

and consequently, we get

A13 $$\begin{aligned} C_2= \frac{b}{|\mathfrak {S}|}\frac{\Gamma \left( \frac{\alpha }{\alpha -1}+\frac{Q}{b}\right) }{\Gamma (\frac{\alpha }{\alpha -1})\Gamma (\frac{Q}{b})}\,. \end{aligned}$$

Following the same arguments as before one can compute the $L^{\alpha }(\mathbb {G})$-norm of $\phi _2$ and get

A14 $$\begin{aligned} \Vert \phi _2\Vert _{L^{\alpha }(\mathbb {G})}^{\alpha }=C_{2}^{\alpha -1}\frac{\alpha b }{b \alpha +Q(\alpha -1)}\,. \end{aligned}$$

## Declarations

### associated data

All three authors have equally contributed. The manuscript has no associated data.
